# Design of UV-Resistant Polylactide-Based Coating Systems: Effect of Plasticizers and Fillers on Durability and Degradation Behavior

**DOI:** 10.3390/ma19122520

**Published:** 2026-06-11

**Authors:** Oleksiy Myronyuk, Denys Baklan, Myroslav Domashevskyi, Taras Karavayev, Olena Sevastyanova

**Affiliations:** 1 Department of Chemical Technology of Composite Materials, Chemical Technology Faculty, Igor Sikorsky Kyiv Polytechnic Institute, Beresteiskyi Ave. 37, 03056 Kyiv, Ukraine; o.myronyuk@kpi.ua; 2 Department of Commodity Science and Customs Affairs, State University of Trade and Economics, Kyoto St., 19, 02156 Kyiv, Ukraine; m.domashevskyy@knute.edu.ua (M.D.); t.karavayev@knute.edu.ua (T.K.); 3 Division of Wood Chemistry and Pulp Technology, Department of Fiber and Polymer Technology, KTH Royal Institute of Technology, Teknikringen 56–58, 100 44 Stockholm, Sweden

**Keywords:** polylactide, polymer, durability, degradation, plasticizer, filler, polymer film

## Abstract

Polylactic acid (PLA) is a promising biopolymer for environmentally friendly coating development. However, its UV radiation resistance has not yet been sufficiently studied, particularly in formulations containing plasticizers or fillers. In this study, a series of samples were prepared: pure PLA films, PLA films with plasticizers, filled composites, and films obtained from aqueous PLA dispersions. The samples were tested for UV resistance and characterized using FTIR spectroscopy, surface energy analysis, and topography. The results showed that UV irradiation of pure PLA caused carbonyl band broadening and a shift toward lower wavenumbers, water contact angle decrease and surface energy polar component increase. The effect of plasticizers was chemical composition-dependent; epoxy linoleic acid increased the degradation rate, whereas PEG-400 and menthol oleic acid reduced the carbonyl groups accumulation. Menthol oleic acid demonstrated the strongest stabilizing effect. The calcite and kaolin fillers promoted surface oxidation and hydrophilization, while coffee grounds biochar reduced the degradation rate. Films obtained from aqueous dispersions were the most sensitive to UV aging, as residual emulsifier significantly enhanced surface hydrophilization.

## 1. Introduction

In recent years, bio-based and biodegradable polymers have attracted increasing attention due to environmental concerns and the need for sustainable material alternatives. This interest is reflected in the rapidly growing body of literature on the synthesis, properties, and degradation mechanisms of these materials [1]. Polylactide (PLA) is among the most widely studied and industrially relevant biodegradable polymers [2].

A substantial body of research has been devoted to polylactic acid (PLA), primarily focusing on bulk materials for applications such as additive manufacturing and conventional plastics, as well as films for packaging applications [3,4]. PLA has been extensively investigated as a sustainable alternative for food packaging films due to its biodegradability and favorable processing characteristics [5]. However, significantly less attention has been given to PLA in the form of functional coatings, especially regarding formulation design and long-term performance. Studies on PLA-based coatings are limited, often focusing on specific applications rather than providing a comprehensive understanding of structure–property relationships [6]. UV-protective properties of biodegradable polymer films and the use of aromatic bio-based fillers for UV absorption have also been addressed recently in related works [7,8].

A closely related yet separate issue concerns the intrinsic biodegradability of polymers such as polylactic acid and polyhydroxyalkanoates. While this characteristic is generally considered a significant advantage from a sustainability standpoint, it also poses challenges when it comes to evaluating their long-term durability under real-world conditions. In this context, biodegradability and durability are inherently antagonistic, as a material’s capacity to undergo degradation may compromise its ability to maintain stable performance over time [9]. This issue underscores the importance of systematically investigating the aging processes of biopolymer-based composites, particularly with respect to performance stability and structural integrity over time. The authors of [10] attempted to compare the biodegradation speed and UV stability of PLA blends with different biodegradable polymers. One study [11] reports the effect of UV-accelerated aging on the mechanical properties of injection-molded PLA-hemp fiber composites. Another study [12] determined the effect of reinforcing fiber surface treatment on the long-term durability of pressed composites. These studies clearly demonstrate the relationship between a composite’s composition and its stability under accelerated aging conditions. This relationship is the basis for engineering durable materials. However, these studies primarily focus on a set of target strength properties characteristic of plastics. These properties are not the determining factors for thin-film coatings.

The photodegradation of PLA polymers is described as a sequence of photooxidative reactions started with a cleavage of ester bonds. It was shown [13] that direct photolysis and subsequent Norrish I and II reactions produce low molecular weight products [14] with carbonyl, hydroxyl, carboxyl and unsaturated functional groups leading to the increase in the surface polarity of the specimen—that means hydrophilization. Although these mechanisms have been extensively investigated for bulk PLA, their manifestation in thin coating systems may differ due to the high surface-to-volume ratio and the presence of formulation additives capable of modifying oxidation pathways, degradation kinetics, and surface reconstruction processes during aging.

When designing PLA-based coating systems, it is important to consider the role of additives, as they are essential for tailoring material performance. Two key classes of additives that critically influence both functional properties and long-term stability are fillers and plasticizers. Fillers are commonly introduced to enhance mechanical strength, improve barrier properties, and provide protective effects, such as UV shielding and light scattering. These effects can potentially mitigate photoinduced degradation processes [15,16]. Conversely, plasticizers are widely used in PLA systems to improve flexibility and processability by increasing polymer chain mobility and reducing brittleness [17]. However, this modification may affect the material’s stability under environmental exposure because increased molecular mobility can accelerate degradation processes. This was illustrated in [18] using PLA-oligomeric lactic acid blends as an example. Therefore, the combined effect of fillers and plasticizers is crucial in determining the performance and durability of PLA-based coatings, making these components important targets for investigation.

Despite the extensive research on PLA-based materials and the well-documented influence of fillers and plasticizers on their mechanical and functional properties, the combined impact of these factors on the durability of PLA in coating applications is not well understood. Specifically, the mechanisms that govern UV-induced degradation in plasticized and filled PLA-based coatings, as well as the resulting structure–property–durability relationships, are not yet fully understood.

This study aims to systematically investigate the effects of fillers and plasticizers on the UV-induced degradation of PLA-based coatings, particularly their influence on surface chemistry, wettability, and morphological evolution. This study also seeks to establish the relationships between the structure, properties, and durability that govern the performance of these systems under UV exposure.

## 2. Materials and Methods

### 2.1. Materials

In this work as polylactide (PLA) the Ingeo Biopolymer 4060D (NatureWorks, Minnetonka, MN, USA) was used. As a solvent for PLA dichloromethane (Carlo Erba, Peypin, France) was used to form 5 wt. % PLA solution. The plasticizers used were polyethylene glycol (PEG-400) (as reference [17]) (HLR Ukraine, Chemlaborreactiv LLC, Brovary, Ukraine), epoxy linoleic acid (synthesized according to the methodology described in previous paper [19]) and deep eutectic solvent menthol–oleic acid 1:1 (MenOl). The fillers used were ground calcite (Normcal 2, Som Calcite, Ankara, Turkey) with D_50_ = 2.1 µm, kaolin (Sanblend 90, Antwerpen, Belgium, Sibelco) with D_50_ = 0.9 µm and coffee grounds biochar with D_50_ = 5.5 µm. Coffee grounds biochar was produced through a thermal treatment process (1 h at 400 °C) of coffee grounds obtained from local cafes as waste material.

### 2.2. Obtaining Polylactide Dispersions

PLA dispersions were prepared following the procedure described in detail in [20], using sodium dodecyl sulfate (SDS) (HLR Ukraine, Chemlaborreactiv LLC, Brovary, Ukraine) as emulsifier, mechanical dispersion followed by ultrasonication, and solvent removal by rotary evaporation.

### 2.3. Obtaining of Films and UV Aging

The compositions were prepared by mixing the PLA solution and plasticizers using a top-drive mixer (OS-20, Riva Steel, Kyiv, Ukraine). The plasticizer and fillers concentration was 20 wt. % relative to the PLA mass (20 g of plasticizer per 100 g of PLA). A high-speed disperser (WiseTis HG 15A, Daihan Scientific, Daejeon, Republic of Korea) was used to introduce fillers and completely break up agglomerates. All fillers were pre-dried. The resulting compositions were applied to steel substrates using an applicator. The coating thickness was 30 µm.

Solution-processed PLA films were used as a reference for comparing the rate and nature of aging in composite films. The thickness of these films was maintained within 30 µm, and the solvent (dichloromethane) was evaporated after application during 48 h of exposure under a fume hood and subsequently in a drying oven at 50 °C.

UV aging tests were conducted in a UV chamber (BGD 852, Biuged Precision Instruments, Guangzhou, China) equipped with UV-C lamps (maximum emission wavelength of 254 nm). The irradiance at the sample surface was 6 mW/cm^2^, measured at a lamp-to-sample distance of 11 cm. Water misting was turned off to minimize its influence. The temperature during the test was maintained at 60 °C. UV-C lamps were used as an accelerated screening source; it is worth mentioning that absolute degradation rates and some reaction pathways may differ under UV-A/B irradiation representative of outdoor conditions (ISO 4892-3) [21]. The purpose of UV-C irradiation was not to reproduce outdoor weathering conditions directly, but to accelerate photooxidative degradation and enable comparison between formulations within a practical experimental time frame.

### 2.4. Characterization

Chemical analysis was performed via FTIR spectroscopy in ATR mode (IRSpirit, Shimadzu, Kyoto, Japan). For water, water–isopropanol mixtures, isopropanol, and cyclohexane, surface tension values were obtained using the pendant drop technique (BGD-190, Biuged Precision Instruments, Guangzhou, China). Contact angles for different liquids were obtained using contact angle goniometer (BGD-190, Biuged Precision Instruments, Guangzhou, China). A 1 mL syringe was used to dispense the 5 µL drop volume. The droplets were applied at 10 different positions on the sample surface. After measuring the contact angles, the samples were dried at 30 °C for 10 min. The PLA film surface free energy was calculated from the contact angles values of test liquids using the Owens–Wendt approach, which separates the total surface energy (σ) into dispersive (σ^D^) and polar (σ^P^) components.

FTIR spectra were processed in OriginPro software (ver. 2024, OriginLab Corporation, Northampton, MA, USA). Prior to peak area analysis, all spectra were baseline-corrected (asymmetric least squares smoothing). The carbonyl stretching region was then deconvoluted using the same fitting procedure for all samples (Gaussian method), and the areas of the fitted carbonyl peaks were calculated [13].

The surface topography was studied using a scanning electron microscopy in SE mode (MIRA3 LMU, Tescan, Brno, Czech Republic). The samples were coated with a 30 nm layer of gold–platinum to reduce the surface charge (682 PECS, Gatan, Inc., Pleasanton, CA, USA).

Hansen solubility parameters (HSP) were determined using a set of solvents (water, dichloromethane, isopropyl alcohol, hexane, ethyl acetate, n-butanol, cyclohexane, dimethylformamide, o-xylene, and dimethyl sulfoxide) or their mixtures. All named substances were purchased from the local supplier HLR Ukraine (Chemlaborreactiv LLC, Brovary, Ukraine). The dichloromethane was used as the solvent for PLA. According to the Hansen model, the radius ratio between the plasticizer (R_a_) and polymer (R_0_) spheres is a condition for their compatibility. The Relative Energy Difference (RED) parameter is the ratio of R_a_/R_0_. For RED > 1, the plasticizer is incompatible with the polymer and if RED < 1, it is compatible. The solubility borders and sphere center of PLA in D, P, and H coordinates were calculated using the HSP Excel spreadsheet developed by Dr. Diaz de los Rios [22].

## 3. Results and Discussion

### 3.1. Degradation of Neat PLA Polymer Film

**Figure 1:**
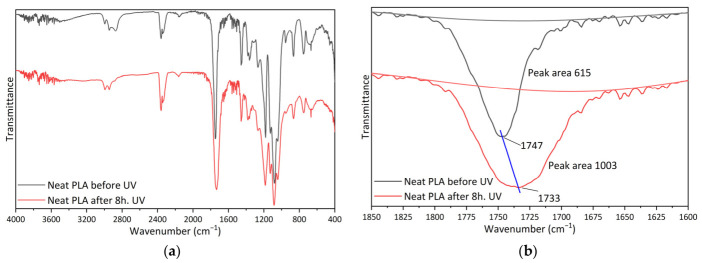
FTIR spectra evolution of neat PLA after 8 h UV exposure: (**a**) the 4000–400 cm^−1^ spectrum; (**b**) region of carboxylic stretching vibration band. The blue line shows the change in the peak value.

Polylactide films were subjected to artificial aging in a UV chamber for 8 h, and the chemical changes in their composition were studied using FTIR (Figure 1).

The PLA FTIR spectrum shows peaks corresponding to the symmetric and asymmetric valence vibrations of the C–H groups, which fall within the 2995–2848 cm^−1^ range. There is a high-intensity peak at 1747 cm^−1^ from the carbonyl group of the ester. Additionally, there are CH_3_ bending vibrations in the range of approximately 1456–1363 cm^−1^ and a set of intense bands in the range of 1267–1044 cm^−1^ corresponding to C–O–C and C–O vibrations in the PLA ether group.

After UV irradiation, the positions and set of the main bands generally remain unchanged, indicating that the degradation was only partial. However, their shape and intensity change. The most noticeable changes occur in the region of the carbonyl peak at 1733 cm^−1^. There is an increase in peak width and a redistribution of peak intensity, which is associated with the appearance of PLA degradation products. For initial PLA, the maximum of the carbonyl band occurs at 1747 cm^−1^. After UV irradiation, the maximum shifts to the low-frequency region at 1733 cm^−1^. Since the absorption bands overlap, directly calculating the carbonyl index based on the ratio of C=O and C-H band intensities, as is done with polyolefins, is difficult [23]. Therefore, this study uses a method based on the ratio of carbonyl peak areas before and after degradation [24]. For pure PLA, the peak area increases from 615 units before UV irradiation to 1003 units after UV irradiation. To compare the extent of the degradation processes based on the ratio of these peak areas in this work, we use the coefficient Ka = S1747/S1730. The reference value of Ka for pure polylactide is 1.63. A shift in the absorption band maximum toward lower w-values indicates that the carbonyl groups are in a new chemical environment. An increase in the band area indicates an increase in the total content of carbonyl-containing groups in the sample.

These results are consistent with previous findings, particularly with regard to the characteristic evolution of the PLA carbonyl region during UV exposure. Notably, broadening of the shoulder of the main peak at 1747 cm^−1^ toward lower wavenumbers is observed [25]. According to the currently accepted mechanism of PLA photodegradation, UV irradiation excites ester carbonyl groups and initiates Norrish type I and type II reactions. Norrish type I reactions involve bond cleavage adjacent to the carbonyl group, leading to chain scission and the formation of lower-molecular-weight fragments. Norrish type II reactions proceed through intramolecular hydrogen abstraction followed by rearrangement reactions, generating unsaturated structures and additional oxygen-containing species. The combined action of these pathways results in the accumulation of carbonyl-containing degradation products in different chemical environments, which is reflected in the broadening and low-wavenumber shift in the carbonyl absorption band. Ultimately, these processes lead to the formation of additional unsaturated, carbonyl-, carboxyl-, and hydroxyl-containing species, accompanied by a decrease in molecular weight, as previously reported in [13]. In practice, this results in the formation of a polar surface layer with increased affinity toward water. Additionally, polylactide is effectively plasticized by its own oligomers [26]. This effect is likely also relevant for the partially oxidized low- and medium-molecular-weight products formed during photodegradation.

**Figure 2:**
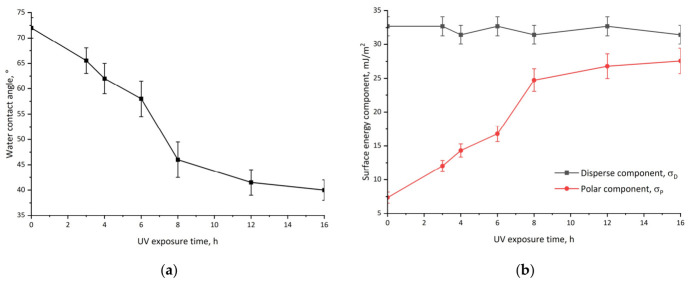
PLA film surface properties change during UV degradation: (**a**) water contact angle change; (**b**) surface energy components evolution.

The accumulation of carboxyl groups increases the potential of the surface for polar and hydrogen bonding interactions, resulting in improved wetting by water (Figure 2).

This hydrophilization is accompanied by a decrease in the contact angle from 72° to 45°, which occurs within the first eight hours. Thereafter, this value remains relatively constant. Wetting increases due to growth in the polar component of the PLA film’s surface energy to nearly 27 mJ/m^2^ (Figure 2b). However, the dispersive component remains virtually unchanged during oxidation, staying at the neat PLA level of 30–32 mJ/m^2^. Similar changes in total surface tension have been noted in previous studies [27,28].

The use of polymer solutions to produce coatings goes against the principles of green chemistry due to the volatility of solvents. This study examined the behavior of films obtained from polylactide dispersions in water using the method previously described [19]. Since surfactants (in this case, sodium dodecyl sulfonate) are used to stabilize the particles when preparing the dispersions, traces of the surfactants are deposited on the surface during film formation. This results in water contact angles of 32 ± 3° for such surfaces. However, this hydrophilic layer can be removed by washing the surface with water. After drying, the water contact angle returns to its initial value of 70 ± 2°. When the UV film obtained from the dispersion is exposed without washing, the contact angle remains at 32 ± 5° throughout the entire 16 h test.

**Figure 3:**
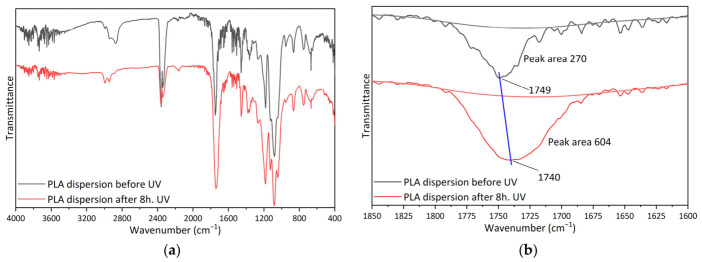
FTIR spectra evolution of PLA film obtained from dispersion after 8 h of UV exposure: (**a**) the 4000–400 cm^−1^ spectrum; (**b**) region of carboxylic stretching vibration band. The blue line shows the change in the peak value.

Exposure to UV light of these films obtained from water dispersion results in an increase in the number of surfaces in oxygen-containing polar groups similar to that of neat PLA processes (Figure 3). However, it is worth mentioning that, in this case, the SDS layer undergoes photooxidation along with the polymer. Note that the Ka is 2.23 in this case, which clearly indicates that the surface is more vulnerable to polarization than the surface of a solvent-based film.

The aging of films obtained from dispersions is thus influenced by traces of surfactants, and these traces may remain trapped within the coating between fused dispersion particles after film formation [29,30].

Further on in this work, we examine the influence of plasticizers and fillers as the most important macrocomponents of modern coatings on the aging of polylactide films under UV irradiation.

### 3.2. The Role of Plasticizers

**Figure 4:**
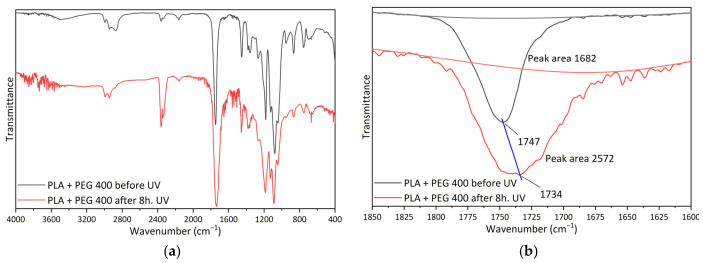
FTIR spectra evolution of PLA + PEG 400 film after 8 h of UV exposure: (**a**) the 4000–400 cm^−1^ spectrum; (**b**) region of carboxylic stretching vibration band. The blue line shows the change in the peak value.

**Figure 5:**
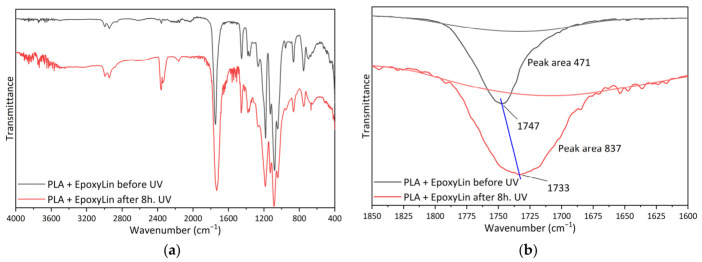
FTIR spectra evolution of PLA + Epoxy linoleic acid film after 8 h of UV exposure: (**a**) the 4000–400 cm^−1^ spectrum; (**b**) region of carboxylic stretching vibration band. The blue line shows the change in the peak value.

**Figure 6:**
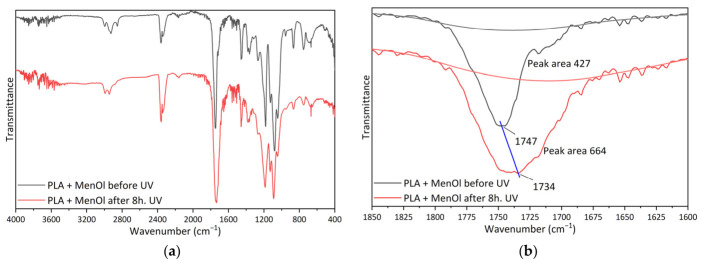
FTIR spectra evolution of PLA + Menthol oleic acid after 8 h of UV exposure: (**a**) the 4000–400 cm^−1^ spectrum; (**b**) region of carboxylic stretching vibration band. The blue line shows the change in the peak value.

The FTIR spectra after 8 h of UV irradiation show a similar trend to that of unmodified PLA. Following UV exposure, a shift in the carbonyl band to the 1733–1734 cm^−1^ region is observed, as well as an increase in peak area. The Ka coefficient for the PLA sample plasticized with PEG-400 (Figure 4) is 1.52, which is lower than neat PLA. For PLA-EpoxyLin (Figure 5), the ratio is slightly higher at 1.77, and for PLA-MenOl (Figure 6), it is the lowest at 1.4.

As with the SDS example it is expected that plasticizer molecules will participate in the photooxidation process (Figure 5). Their photooxidation products may contribute to the surface state and configuration of the absorption band around 1740 cm^−1^ or be removed if the products formed are volatile. This well-known effect is described in references [31,32].

Considering the low concentration of the plasticizer relative to the polymer and the screening of its absorption bands, it is difficult to determine the fate of the plasticizer molecules from the FTIR spectra. However, by comparing the areas of the absorption bands corresponding to the C=O stretching vibration, we can conclude that the plasticizers PEG-400 and Menthol oleic acid reduce surface carbonation (Figure 6), while the Epoxy linoleic acid plasticizer slightly increases it during UV exposure.

**Figure 7:**
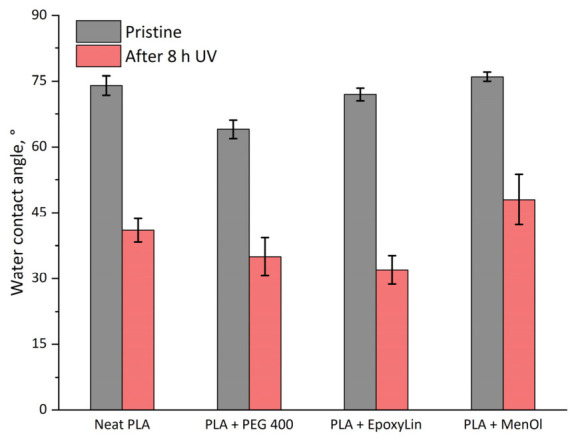
Water contact angle change during UV exposure for plasticized PLA films.

Note that wettability depends on the type of plasticizer (Figure 7). For the plasticizers under consideration, wettability generally decreases (Epoxy linoleic acid and PEG 400). However, a slight increase in the contact angle is observed for the hydrophobic Menthol oleic acid. During photoaging, the contact angles decrease to a minimum of 32° for Epoxy linoleic acid. Menthol oleic acid is the most stable, maintaining a value of 48°.

The surface energy changes accordingly (see Table 1). The initial σ_0_ values for the films are practically identical within the margin of error. However, significant differences can be observed in the σ^P^ values. The highest initial value is found in the PLA + PEG 400 film. However, due to photooxidation, the EpoxyLin plasticized film surface becomes the most polarized, while MenOl exhibits the greatest stability.

In addition to changes in surface energy, the material’s solubility parameters also change; since UV oxidation changes the polarity and chemical structure of PLA, the HSP analysis was used to estimate whether the affinity between PLA and the plasticizers changes after irradiation. This aspect is important because it reflects changes in the compatibility between polylactic acid (PLA) and the plasticizer. To investigate this, we exposed a PLA film sample to UV light for eight hours to obtain a sufficient amount of oxidized material to determine its Hansen parameters. Table 2 presents the HSPs for the PLA polymer in its initial and oxidized states, as well as for the initial plasticizers and for plasticizers in UV-aged PLA polymer. It also presents the RED value, which indicates the affinity grade.

Thus, the material formed during oxidation differs significantly from the original polymer, and the solubility center shifts from (16.5, 9.9, 6.40) to (16.64, 11.35, 4.66) primarily due to an increased ability to form polar van der Waals interactions during oxidation.

Although UV oxidation of PLA is expected to generate hydroxyl and carboxyl groups, which normally increase hydrogen-bonding interactions, the observed decrease in δH may result from reduced accessibility of these groups due to chain scission-induced structural rearrangement and partial crystallization of low-molecular-weight fragments. Such reorganization can limit specific solvent–polymer hydrogen bonding despite the overall increase in polarity reflected by the higher δP value. In addition, the limited number of probe solvents used for HSP fitting may contribute to uncertainty in the calculated δH values for oxidized PLA surfaces. The solubility sphere expands by 8.5% in radius (R_0_). Consequently, the RED index, which describes the compatibility between the polymer and the plasticizer, increases as well. According to Hansen’s theory, RED > 1 indicates incompatibility; however, the boundary of compatibility is unclear, and partial incompatibility can be observed at values close to unity [32]. According to our observations, partial incompatibility begins around a RED value of 0.8. The plasticizers PEG-400 and menthol–oleic acid 1:1 reach these values, which may cause phase separation and “migration” of the plasticizer to the film surface.

It can be assumed that the PLA + MenOl film is susceptible to plasticizer migration, which increases the plasticizer content in the upper layers and decreases its polarity. However, the FTIR and surface energy data indicate a reduction in PLA photooxidation speed in the presence of this plasticizer. In contrast, EpoxyLin seems to be compatible with PLA, even after UV exposure. However, it noticeably increases wettability. To prove that the plasticizer migrates, the top layer of the substance was removed using isopropyl alcohol. As a result, the water contact angle increased to 35° for PLA + PEG-400, 34° for PLA + EpoxyLin sample and 58° for PLA + MenOl film.

The three plasticizers also differ markedly in their plasticization efficiency: our previous work, [20], reports T_g_ depressions of −17 °C for PEG-400, −10 °C for epoxidized linoleic acid, and −6 °C for MenOl at the same 20 wt. % loading relative to the PLA mass. Comparing these values with the Ka results reveals an inverse relationship between plasticization efficiency and UV stability: the weakest plasticizer (MenOl, −6 °C) affords the best UV protection (lowest Ka = 1.40), while the strongest plasticizer (PEG-400, −17 °C) gives only moderate protection (Ka = 1.52). This trend suggests that higher chain mobility—induced by more effective plasticization—facilitates faster diffusion of reactive oxygen species and degradation intermediates into the bulk polymer, thereby accelerating photodegradation.

However, this approach is limited by the fact that RED is calculated for the original plasticizers rather than their photodegradation products. For a more comprehensive future model of compatibility changes, this factor, as well as the kinetics of plasticizer degradation into simple compounds, should be considered.

The observed behavior suggests that the UV-aging response of plasticized PLA is controlled by the interplay between plasticization-induced chain mobility and compatibility changes arising during oxidation, both of which can influence degradation kinetics and surface composition. 

### 3.3. The Role of Fillers

Unlike plasticizers, which are distributed at the molecular or cluster level within the PLA polymer, fillers are dispersed particles that form a phase boundary with the polymer matrix. These particulate materials, particularly inorganic kaolin and calcite, are more resistant to photodegradation than the surrounding PLA.

**Figure 8:**
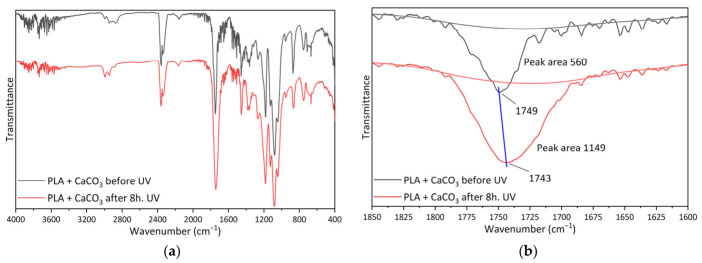
FTIR spectra evolution of PLA + Calcite film after 8 h of UV exposure: (**a**) the 4000–400 cm^−1^ spectrum; (**b**) region of carboxylic stretching vibration band. The blue line shows the change in the peak value.

As can be seen in Figure 8, the calcite Ka value is 2.05, the highest among the systems considered. It is important to note that for CaCO_3_, the high Ka value for the calcite composite is attributed to the high specific surface area of CaCO_3_ particles, which increases the effective UV-irradiated polymer volume and may promote catalytic ester bond cleavage at the particle–polymer interface [33].

**Figure 9:**
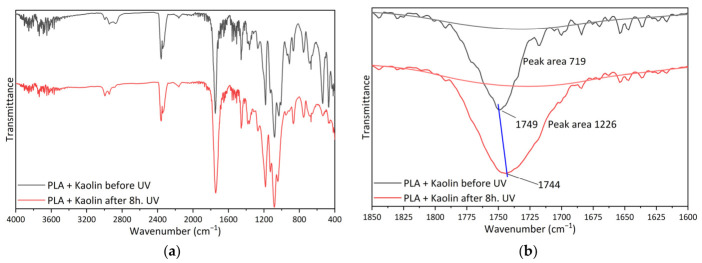
FTIR spectra evolution of PLA + Kaolin film after 8 h of UV exposure: (**a**) the 4000–400 cm^−1^ spectrum; (**b**) region of carboxylic stretching vibration band. The blue line shows the change in the peak value.

The kaolin sample (Figure 9) is far more stable, with a Ka value of only 1.7, slightly higher than neat PLA. This filler does not contain C=O bonds, so even if its particle surface is exposed, it should lead to a decrease in the intensity of the respective carboxylic bands.

The interfacial chemistry of mineral fillers can influence the degradation behavior of the surrounding polymer matrix. Calcite particles possess surface hydroxyl groups and basic sites that may promote ester bond cleavage [34] and facilitate the formation of degradation products within the polymer–filler interphase. Furthermore, the high specific surface area of CaCO_3_ increases the fraction of polymer located in the interfacial region, where degradation processes may differ from those occurring in the bulk material. These factors may contribute to the higher degradation coefficient observed for the calcite-filled system. In contrast, the lower activity observed for kaolin-filled composites suggests a weaker influence of the filler surface on degradation reactions.

**Figure 10:**
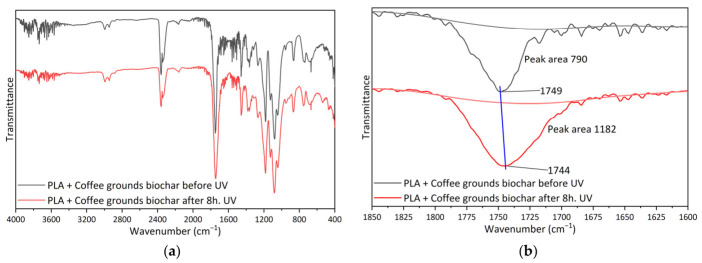
FTIR spectra evolution of PLA + coffee grounds biochar film after 8 h of UV exposure: (**a**) the 4000–400 cm^−1^ spectrum; (**b**) region of carboxylic stretching vibration band. The blue line shows the change in the peak value.

Unlike the mineral-filled composite, the coffee ground biochar composite (Figure 10) shows a reduction in Ka from 1.63 to 1.49. However, the mechanism of this reduction is not evident. It is known that the oxidation of this filler can lead to the formation of additional oxygen-containing polar groups [35]. On the other hand, the condensed aromatic structures in coffee-ground biochar may act as UV absorbers, analogously to the UV-blocking role of aromatic lignin structures reported for polymer coatings [7]. Additionally, biochar particles can absorb UV radiation due to their carbonized nature. This shielding effect reduces photoinduced reactions in the polymer matrix, thereby slowing oxidative degradation and carbonization processes in the bulk material. Aromatic carbon domains present in biochar can absorb UV radiation and dissipate the absorbed energy through non-radiative relaxation pathways, thereby reducing the photon flux reaching the surrounding PLA matrix.

These differences in photodegradation behavior are accompanied by changes in surface polarity (Table 3). Calcium carbonate exhibits the most intense polarization and an increase in the polar component of surface energy.

The coffee ground biochar had the lowest increase, but its polar surface energy component is still higher than that of the neat PLA film. Unlike plasticizers, which can migrate to the surface due to incompatibility with the degraded matrix, the fillers remain on the surface due to oxidation and the removal of the upper matrix layer. The initial inorganic fillers have hydrophilic surfaces [36,37], and the biochar has a hydrophobic surface due to contamination from aromatic hydrocarbon pyrolysis products, for example [38] reports a water contact angle value of 126°.

**Figure 11:**
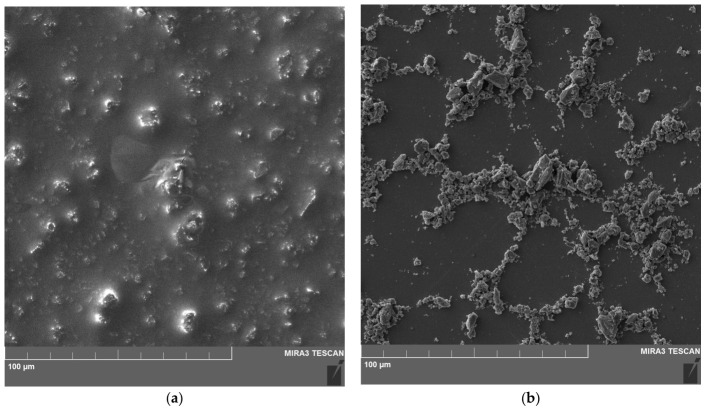
The surface of PLA film filled with calcite after UV exposure: (**a**) after 4 h; (**b**) after 16 h.

As shown in Figure 11, when the surface of composite films is exposed to UV light, the proportion of the polymer binder gradually decreases. In Figure 11a, which corresponds to 4 h of oxidation, the partially oxidized matrix envelops the particles, though some of their surface is exposed. In Figure 11b, which corresponds to 16 h of testing, the matrix layer is no longer discernible. The final figure shows that the particles have aggregated into clusters, likely due to capillary effects occurring during the decomposition of the liquid products prior to their final ablation.

**Figure 12:**
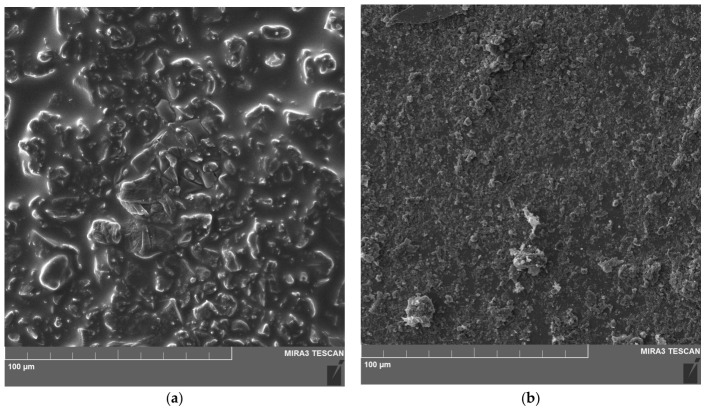
The surface of PLA composite films after 8 h UV exposure: (**a**) filled with ground coffee biochar; (**b**) filled with kaolin.

Systems filled with kaolin and biochar coffee grounds behave similarly. The matrix burns out quickly, exposing the surface of the filler particles (Figure 12a). As seen in Figure 12b, the fine kaolin particles are exposed more than the large biochar particles. However, the exposed particle surfaces in the lower left corner of Figure 12a exhibit a noticeable substructure, indicating the absence of a significant amount of matrix on their surfaces.

In general, the degradation of filled composites involves the simultaneous occurrence of hydrophilization and depolymerization of the polymer matrix. This process also involves the removal of volatile products and the exposure of filler particle surfaces. These surfaces likely remain coated with hydrophilic polymer degradation products until a certain point. The results indicate that the effect of fillers on PLA photodegradation is controlled by both interfacial interactions and morphological evolution of the composite surface, as progressive matrix degradation exposes filler particles that can further modify wettability, surface polarity, and degradation behavior.

An exposed, hydrophilized surface acts as a capillary body. When measuring wetting properties, this leads to distortions in the contact angle values described by the Wenzel equation (primarily a decrease in the case of hydrophilic materials).

Comparing the contributions of fillers, plasticizers, and emulsifiers to the hydrophilization of the polymer matrix (Table 4) reveals that these classes can accelerate or slow down degradation and hydrophilization depending on the composition. The most effective accelerators of these processes are the emulsifier (SDS), calcite, and the epoxy-linolenic acid plasticizer. Coffee ground biochar and MenOl act as retardants, though their relative hydrophobicity cannot serve as a universal explanation for their effect.

Thus, it can be concluded that the effect of additives on the degradation process is complex. In the case of plasticizers, this effect is determined by factors such as the polarity of the starting component, its sensitivity to irradiation, and its compatibility with the matrix. For example, MenOl may migrate to the surface, protecting the main polymer from degradation (or distorting the measurement data due to its non-polarity relative to PLA). For fillers, surface polarity and the ability to inhibit or initiate reactions in degradation processes play a role. In this sense, the slight UV stabilization provided by aromatic compounds in coffee ground biochar significantly slows down degradation.

## 4. Conclusions

Thus, based on the results of the analysis of the photodegradation processes of PLA-based composites using FTIR spectroscopy, surface energy characterization, scanning electron microscopy (SEM), and contact angle determination, the following have been established:

The epoxy linoleic acid-based plasticizer accelerates the photodegradation process (Ka increases by 8.5% compared to the initial polymer), whereas polyethylene glycol and MenOl reduce its rate (Ka decreases by 6.7% and 14.0%, respectively). Furthermore, an inverse relationship was observed between plasticization efficiency and UV stability: MenOl, the weakest plasticizer in terms of Tg depression (−6 °C), provided the strongest UV stabilization (lowest Ka = 1.40), while PEG-400, the most effective plasticizer (−17 °C), offered only moderate protection (Ka = 1.52). This suggests that higher chain mobility induced by stronger plasticization facilitates oxidative degradation.

Mineral fillers, such as calcite and kaolin, generally accelerate photodegradation. The increase in Ka is significant for calcite (26.5%) and nearly imperceptible for kaolin (4.3%). During photodegradation, the particle surface is exposed, leading to increased wettability. In contrast, coffee ground biochar reduces this value by 8.5%. Considering that its particles are also exposed, it can be considered an effective degradation inhibitor.

A significant challenge for UV resistance is the presence of an emulsifier. During film formation, the emulsifier migrates to the surface of the coating, causing strong hydrophilization and reaching a Ka value of 2.23.

## Figures and Tables

**Table 1:** Surface energy (mJ/m^2^) of pristine and UV-aged, plasticized PLA films.

Sample	Before UV			After UV		
	σ	σ^D^	σ^P^	σ	σ^D^	σ^P^
Neat PLA	40.06 ± 0.88	32.70 ± 0.72	7.36 ± 0.16	59.01 ± 1.56	31.45 ± 0.84	27.56 ± 0.74
PLA + PEG 400	43.63 ± 0.90	29.73 ± 0.61	13.90 ± 0.29	61.72 ± 2.68	28.57 ± 1.24	33.15 ± 1.44
PLA + EpoxyLin	41.32 ± 0.59	33.15 ± 0.48	8.17 ± 0.12	63.31 ± 2.07	28.00 ± 0.92	35.31 ± 1.16
PLA + MenOl	39.87 ± 0.41	33.71 ± 0.35	6.15 ± 0.06	53.88 ± 3.08	29.73 ± 1.70	24.15 ± 1.38

**Table 2:** Hansen solubility parameter for PLA polymer before and after destruction and plasticizers.

Sample	ẟD	ẟP	ẟH	R_0_	R_a_	RED
PLA 4060D before UV exposure	16.50	9.90	6.40	8.50		
Epoxy linoleic acid	16.60	11.40	10.50		4.37	0.51
PEG-400	14.60	7.50	9.40		5.40	0.64
Menthol–oleic acid 1:1	16.80	5.00	9.40		5.78	0.68
PLA 4060D after UV exposure	16.64	11.35	4.66	9.23		
Epoxy linoleic acid	16.60	11.40	10.50		5.84	0.63
PEG-400	14.60	7.50	9.40		7.35	0.80
Menthol–oleic acid 1:1	16.80	5.00	9.40		7.93	0.86

**Table 3:** Surface energy (mJ/m^2^) of pristine and UV-aged, filled PLA films.

Sample	Before UV			After UV		
	σ	σ^D^	σ^P^	σ	σ^D^	σ^P^
Neat PLA	40.06 ± 0.88	32.70 ± 0.72	7.36 ± 0.16	59.01 ± 1.58	31.45 ± 0.84	27.56 ± 0.74
PLA + CaCO_3_	38.38 ± 1.18	32.59 ± 1.00	5.79 ± 0.18	66.36 ± 3.54	27.42 ± 1.46	38.94 ± 2.08
PLA + Kaolin	40.65 ± 0.99	33.15 ± 0.81	7.50 ± 0.18	63.87 ± 2.73	28.00 ± 1.20	35.87 ± 1.53
PLA + Biochar	40.71 ± 0.83	33.71 ± 0.69	7.00 ± 0.14	59.74 ± 3.42	28.00 ± 1.60	31.74 ± 1.82

**Table 4:** Characteristics of composite degradation (after UV irradiation).

Sample	Ka	σ^P^
Neat PLA	1.63	27.56
PLA + PEG 400	1.52 ↓*	33.15 ↑
PLA + Epoxy Lin	1.77 ↑	35.31 ↑
PLA + MenOl	1.40 ↓	24.15 ↓
PLA + Calcite	2.05 ↑↑	38.94 ↑↑
PLA + Kaolin	1.7 ↑	35.87 ↑
PLA + Biochar	1.49 ↓	31.74 ↑
PLA + Emulsifier	2.23 ↑↑	40.12 ↑↑

* The arrows indicate a decrease (↓), an increase (↑), or a twofold increase (↑↑) in the Ka and σ^P^ parameters.

## Data Availability

The original contributions presented in this study are included in the article. Further inquiries can be directed to the corresponding authors.

## Abbreviations

The following abbreviations are used in this manuscript:

PLA	Polylactide
FTIR	Fourier-transform infrared spectroscopy
RED	Relative Energy Difference
PEG	Polyethylene glycol
SDS	Sodium dodecyl sulfate
SEM	Scanning electron microscopy
MenOl	Menthol oleic acid
UV	Ultraviolet
HSP	Hansen solubility parameters

## References

[1] Dallaev R., Papež N., Allaham M.M., Holcman V. (2025). Biodegradable Polymers: Properties, Applications, and Environmental Impact. Polymers.

[2] Khouri N.G., Bahú J.O., Blanco-Llamero C., Severino P., Concha V.O.C., Souto E.B. (2024). Polylactic acid (PLA): Properties, synthesis, and biomedical applications—A review of the literature. J. Mol. Struct..

[3] Olonisakin K., Mohanty A.K., Thimmanagari M., Misra M. (2025). Recent advances in biodegradable polymer blends and their biocomposites: A comprehensive review. Green Chem..

[4] Plamadiala I., Croitoru C., Pop M.A., Roata I.C. (2025). Enhancing Polylactic Acid (PLA) Performance: A Review of Additives in Fused Deposition Modelling (FDM) Filaments. Polymers.

[5] Swetha T.A., Bora A., Mohanrasu K., Balaji P., Raja R., Ponnuchamy K., Muthusamy G., Arun A. (2023). A comprehensive review on polylactic acid (PLA)—Synthesis, processing and application in food packaging. Int. J. Biol. Macromol..

[6] Cao C., Ahn K., Hong S.J., Kim Y.-T., He Z., Huang H., Wang Z., Lee E., Shim Y. (2025). Spray-coated polylactic acid/polyhydroxyalkanoate biodegradable bioplastic films on paper: A sustainable strategy for enhancing barrier and mechanical properties. Prog. Org. Coat..

[7] Goliszek M., Podkościelna B., Smyk N., Sevastyanova O. (2023). Towards lignin valorization: Lignin as a UV-protective bio-additive for polymer coatings. Pure Appl. Chem..

[8] Goliszek-Chabros M., Smyk N., Xu T., Matwijczuk A., Podkościelna B., Sevastyanova O. (2025). Lignin nanoparticle-enhanced PVA foils for UVB/UVC protection. Sci. Rep..

[9] Glaskova-Kuzmina T., Starkova O., Gaidukovs S., Platnieks O., Gaidukova G. (2021). Durability of Biodegradable Polymer Nanocomposites. Polymers.

[10] Ludwiczak J., Dmitruk A., Skwarski M., Kaczyński P., Makuła P. (2023). UV resistance and biodegradation of PLA-based polymeric blends doped with PBS, PBAT, TPS. Int. J. Polym. Anal. Charact..

[11] Sawpan M.A., Islam M.R., Beg M.D.H., Pickering K. (2019). Effect of Accelerated Weathering on Physico-Mechanical Properties of Polylactide Bio-Composites. J. Environ. Polym. Degrad..

[12] Sánchez M.L., Capote G., Patiño J.P. (2020). Effect of surface treatment of fibers on the accelerated aging of biocomposites. Constr. Build. Mater..

[13] Lomakin S., Mikheev Y., Usachev S., Rogovina S., Zhorina L., Perepelitsina E., Levina I., Kuznetsova O., Shilkina N., Iordanskii A. (2024). Evaluation and modeling of polylactide photodegradation under ultraviolet irradiation: Bio-based polyester photolysis mechanism. Polymers.

[14] Virág Á.D., Tóth C., Molnár K. (2023). Photodegradation of Polylactic Acid: Characterisation of Glassy and Melt Behaviour as a Function of Molecular Weight. Int. J. Biol. Macromol..

[15] Purnama P., Saldi Z.S., Samsuri M. (2024). The Development of Polylactide Nanocomposites: A review. J. Compos. Sci..

[16] Mekonnen K., Fanta G., Tilinti B., Regasa M. (2024). Polylactic Acid Based Biocomposite for 3D Printing: A review. Compos. Mater..

[17] Sun S., Weng Y., Zhang C. (2024). Recent advancements in bio-based plasticizers for polylactic acid (PLA): A review. Polym. Test..

[18] Burgos N., Martino V.P., Jimenez A. (2012). Characterization and ageing study of poly(lactic acid) films plasticized with oligomeric lactic acid. Polym. Degrad. Stab..

[19] Myronyuk O., Baklan D., Bilousova A., Smalii I., Vorobyova V., Halysh V., Trus I. (2025). Plasticized polylactide film coating formation from redispersible particles. AppliedChem.

[20] Baklan D., Vorobyova V., Sevastyanova O., Karavayev T., Myronyuk O. (2026). Sustainable waterborne polylactide coatings enabled by hydrophobic deep eutectic solvents plasticization. Polymers.

[21] (2024). Plastics—Methods of Exposure to Laboratory Light Sources, Part 3: Fluorescent UV Lamps.

[22] De Los Ríos M.D., Belmonte R.M. (2022). Extending Microsoft excel and Hansen solubility parameters relationship to double Hansen’s sphere calculation. SN Appl. Sci..

[23] Celik M., Nakano H., Uchida K., Isobe A., Arakawa H. (2023). Comparative evaluation of the carbonyl index of microplastics around the Japan coast. Mar. Pollut. Bull..

[24] Almond J., Sugumaar P., Wenzel M.N., Hill G., Wallis C. (2020). Determination of the carbonyl index of polyethylene and polypropylene using specified area under band methodology with ATR-FTIR spectroscopy. e-Polym..

[25] Wang X., Chen J., Jia W., Huang K., Ma Y. (2024). Comparing the aging processes of PLA and PE: The impact of UV irradiation and water. Processes.

[26] Avolio R., Castaldo R., Gentile G., Ambrogi V., Fiori S., Avella M., Cocca M., Errico M.E. (2015). Plasticization of poly(lactic acid) through blending with oligomers of lactic acid: Effect of the physical aging on properties. Eur. Polym. J..

[27] Yousefzade O., Jeddi J., Vazirinasab E., Garmabi H. (2018). Poly(lactic acid) phase transitions in the presence of nano calcium carbonate: Opposing effect of nanofiller on static and dynamic measurements. J. Thermoplast. Compos. Mater..

[28] Jeantet L., Regazzi A., Taguet A., Pucci M.F., Caro A.S., Quantin J.-C. (2020). Biopolymer blends for mechanical property gradient 3D printed parts. Express Polym. Lett..

[29] Pagnin L., Wiesinger R., Koyun A.N., Schreiner M. (2021). The Effect of Pollutant Gases on Surfactant Migration in Acrylic Emulsion Films: A Comparative Study and Preliminary Evaluation of Surface Cleaning. Polymers.

[30] Arnold C., Klein G., Maaloum M., Ernstsson M., Larsson A., Marie P., Holl Y. (2010). Surfactant distribution in waterborne acrylic films. Colloids Surf. A Physicochem. Eng. Asp..

[31] Wei X.F., Linde E., Hedenqvist M.S. (2019). Plasticiser loss from plastic or rubber products through diffusion and evaporation. npj Mater. Degrad..

[32] Leite-Barbosa O., De Oliveira M.F.L., De Oliveira M.G., Padilha M.C., Veiga-Junior V.F. (2026). Rapid detection of plasticizer migration from UV-Aged PVC films by DART-HRMS. Rapid Commun. Mass Spectrom..

[33] Vasseur E., Leveuf L., Saux V.L., Bruzaud S. (2025). Prediction of compatibility and chemical structure influencing plasticization of poly(3-hydroxybutyrate-co-4-hydroxybutyrate) (P3HB4HB). Polymer.

[34] Burns K., Wu Y.T., Grant C.S. (2003). Mechanisms of calcite dissolution using environmentally benign polyaspartic acid: A rotating disk study. Langmuir.

[35] Oliveira G.A., Gevaerd A., Mangrich A.S., Marcolino-Junior L.H., Bergamini M.F. (2021). Biochar obtained from spent coffee grounds: Evaluation of adsorption properties and its application in a voltammetric sensor for lead (II) ions. Microchem. J..

[36] Ma Y., Tian P., Bounmyxay M., Zeng Y., Wang N. (2021). Calcium Carbonate@silica Composite with Superhydrophobic Properties. Molecules.

[37] Zheng Y., Wang K., Sun L., Shi H., Zhang X. (2022). Preparation of PFDTS-kaolin/PU superamphiphobic coatings with antibacterial, antifouling and improved durability property. Prog. Org. Coat..

[38] Chen W.H., Du J.T., Lee K.T., Ong H.C., Park Y.K., Huang C.C. (2021). Pore volume upgrade of biochar from spent coffee grounds by sodium bicarbonate during torrefaction. Chemosphere.

